# A comparative study of two porous sponge scaffolds prepared by collagen derived from porcine skin and fish scales as burn wound dressings in a rabbit model

**DOI:** 10.1093/rb/rbz036

**Published:** 2019-11-12

**Authors:** Yufei Shi, Hongjian Zhang, Xin Zhang, Zhan Chen, Dan Zhao, Jun Ma

**Affiliations:** 1 Advanced Biomaterials and Tissue Engineering Center, Huazhong University of Science and Technology, Wuhan 430074, China; 2 Department of Biomedical Engineering, College of Life Science and Technology, Huazhong University of Science and Technology, Wuhan 430074, China

**Keywords:** collagen, wound dressings, wound healing

## Abstract

Collagen is widely used in biomedical applications due to its outstanding properties. In this study, highly porous sponge scaffolds were developed by using porcine skin-derived collagen (PSC) and fish scale-derived collagen (FSC), respectively. The morphology and composition of these PSC and FSC scaffolds were compared. The water uptake ratio of FSC scaffolds reached 47.8, which is 1.7 times of PSC scaffolds. The water vapour transmission rates (WVTR) of PSC and FSC scaffolds were 952.6 ± 55.5 and 1090.9 ± 77.1 g/m^2^/day, which could produce a moist healing environment for wounds. Both scaffolds show non-toxicity to L929 fibroblast cells. The burn wound healing efficiency of these two scaffolds was examined *in vivo* using rabbits. No scars around the wounds were observed after applying PSC and SFC scaffolds. Histopathological studies reveal that the wound treated with PSC and FSC scaffolds showed much better wound recovery than gauze and vaseline gauze groups. It was suggested that FSC scaffolds have great potential as same as PSC to be used as burn wound dressing materials.

## Introduction

The global market for the management of wounds and burns is very huge, including conventional wound dressings and advanced wound dressings [1–3]. Wound care with wound dressings is essential to reduce infection and promote recovery. Generally, wound healing involves four stages including haemostasis, inflammatory, proliferation and remodelling in order [3]. A suitable moist environment around the wounds is important to the regeneration of skin tissues and healing process. Conventional wound dressing such as cotton gauze show limited recovery effects in burn management as they could not provide a suitable moist environment for wound healing [1, 2]. Especially, rising incidence of chronic wounds like diabetic ulcers results in a growth of demand for advanced wound dressings in clinic. Burn wounds also need advanced wound dressings. Burn wounds result in severe inflammatory responses and often cause serious secondary injuries when using conventional wound dressings [3].

In the past two decades, many advanced wound dressings based on the principle of moisture therapy have been developed to treat complex wounds like severe burn wounds [3, 4]. Natural polymers including collagen, alginate and cellulose have been extensively studied, which show advanced properties like biocompatibility, moisture preservation and good vapour transmission properties [1, 2, 5]. It is well-known that collagen I is the main component of extracellular matrix in skin tissues, which accounts for about two-thirds of the dry weight of skin [4, 6]. Collagen can initiate fibroblast formation and accelerate endothelial migration into wound tissue, which plays active roles in healing process [2]. Commercial type I collagen materials are usually prepared by using tissues from bovine and pigs [4, 6]. Porous collagen scaffolds or sponges could be prepared by simply freeze-drying solution containing 0.1–5 w/v% dry mater. Different structures could be controlled by varying the concentration and conditions of freezing and drying. The resultant porous scaffolds can adsorb large amounts of exudate fluid, shield against microbial ingrowth and maintain a clean and moist environment for wound healing [6]. Furthermore, collagen was found to stimulate the growth of cells and regeneration of tissues by manipulating wound biochemistry [4]. The preparation of collagen scaffolds could be carried out by decellularization, which was usually called acellular dermal matrix [7]. Acellular porcine dermal matrix has already been used in clinic, e.g. in extensive deep dermal burns, which could facilitate wound healing [8].

Porcine skin-derived collagen (PSC) has been studied extensively demonstrating outstanding biological properties. PSC is the common source for preparing xenograft due to its high similarity to human skin. Second-degree burns could be well treated using PSC, and scar-free healing could be archived in a short period [9]. Besides skin, PSC matrix could be used to correct intraoral mucosal deficiencies, providing an alternative to an autogenous transplant [10]. However, these PSC materials present a risk of zoonosis. In recent years, fish scale-derived collagen (FSC) has triggered great interest in regenerative medicine dressing due to their rich source, cheap cost and low risk of zoonosis [11]. It was reported that FSC could promote growth of blood and lymphatic vessels under growth factor-free conditions [12]. In addition, FSC could be used to prepare composite scaffolds with graphene oxide and load curcumin, which showed good antimicrobial properties and fast wound healing efficiency [13].

Considering the enormous potential of FSC and PSC, the comparison is of great interest for wound healing applications. Herein, the development of PSC scaffolds and FSC scaffolds was introduced, and their evaluation in treating severe burn wounds in a rabbit model was carried out. At the same time, their morphology, swelling properties and vapour transmission property were compared.

## Materials and methods

### Materials

Fresh grass carp (*Ctenopharyngodon idellus*) scales were purchased from a local market. PSC were provided by Jiangsu Youchuang Biomedical Technology Co., Ltd. Trypsin and pepsin used in the experiments were bought from Sigma-Aldrich. Other reagents provided by local suppliers were chemical grade or above.

### Preparation of PSC and FSC

PSC was prepared according to a decellularizing method [14]. Briefly, the epidermal layer and subcutaneous fat of the pig skin was removed to obtain dermis. Dermis was treated with decellularized fluid composed of 0.25% trypsin. The obtained PSC was then dried, smashed, sterilized, sealed and stored at room temperature for the following experiments.

FSC was prepared as previously described [15]. The scales of grass carp were cleaned by tape water carefully. After cutting into small pieces, the scales were put into 1 M acetic acid solutions to decalcify for 24 h. Acid-soluble collagen in grass carp was obtained after filtration and centrifugation at 8000 rpm for 5 min. The filtrate was added to 0.01 M acetic acid solutions containing 5% pepsin and the solution was stirred for 24 h at room temperature. Pepsin soluble collagen was obtained after filtration and centrifugation at 8000 rpm for 5 min. The two kinds of collagen solution extracted by acid and pepsin were mixed and added an equal volume of 1M sodium chloride solution. After 4 h, the mixture was centrifuged at 8000 rpm for 5 min to get collagen gel. The collagen gel was added to 0.5 M acetic acid solutions and stirred until dissolved. The solution was then dialyzed against 0.1 M acetic acid for 24 h followed by dialysis against ultrapure water for 24 h to get purified collagen gel. The collagen gel was freeze-dried for storage and further experiments.

### Fabrication of wound dressings

The fabrication of wound dressings was modified from the production procedure of Pelnac artificial dermis [16]. PSC was added to 1 mM HCl to form a stable solution with a concentration of 1.5% and stirred at 1000 rpm for 1 h. Then the liquid was quickly poured to the prepared mould, which was transferred to −80°C refrigerator for freeze-drying at once. The mould was a silicone sheet with many cuboid of size 20 mm × 20 mm × 5 mm. The dried sponge was cross-linked in 0.25% glutaraldehyde solution for 24 h and washed with ultrapure water for several times. The PSC scaffolds were obtained by freeze-drying of the sponge. The FSC scaffolds were prepared in the same way as PSC.

### Characterization of PSC and FSC scaffolds

#### Morphology

The morphology of the dried PSC and FSC scaffolds was observed by field emission scanning electron microscopy (SEM; Nova nanoSEM 450, FEI, The Netherlands). In order to improve conductivity, the scaffolds were coated with Au.

#### Porosity

The porosity of scaffolds was measured according to the Archimedes’ principle. The porosity (P) was calculated according to the following formulation:

$$P=\frac{w_{s}^{\prime }-w_{s}}{w_{1}-(w_{2}-w_{s}^{\prime })}\times 100\%,$$

where $w_{1}$ is the weight of pycnometer full of ethanol, $w_{s}$ is the weight of sponge, $w_{2}$ is the weight of pycnometer full of ethanol with sponge soaked in ethanol and $w_{s}^{\prime }$ is the weight of ethanol-soaked sponge. Each experiment was performed no less than three times.

#### Structure

Fourier transform infrared (FT-IR) spectra of the samples were taken on a spectrometer (VERTEX 70, Bruker, Germany) by using grinding mixed pellets with KBr, which were recorded with a resolution of 4 cm^−1^ in the range of 4000–450 cm^−1^ and were averaged from 64 scans at room temperature. Sodium dodecyl sulphate-polyacrylamide gel electrophoresis (SDS-PAGE) was conducted on PSC and FSC samples to determine the molecular weight distribution using the methods reported previously [17, 18].

#### Fluid uptake ability

The porous scaffolds were soaked in phosphate buffered saline (PBS) at room temperature for at least 5 min to reach the equilibrium swelling. Subsequently, the sponge was removed for weighing after gently blotted with a filter paper. Fluid uptake ability (*U*) was calculated according to the following formulation:

$$U=\frac{m_{2}-m_{1}}{m_{1}}\times 100\%,$$

where *m*_1_ is the weight of dried sponge, *m*_2_ is the weight of swollen sponge. Each experiment was performed at least three times.

#### Water-holding capacity

The porous scaffolds were soaked in ultrapure water, and the swollen sponge was kept at 37°C and 35% relative humidity in an incubator. At regular intervals, the scaffolds were removed for weighing. Weight remaining (*R*) was calculated according to the following formulation [19]:

$$R=\frac{m_{t}}{m_{0}}\times 100\%,$$

where *m*_0_ is the weight of swollen sponge and *m_t_* is the weight of sponge regular time (*t*) later.

#### Water vapour transmission rate

The moisture permeability of the scaffolds was evaluated by the water vapour transmission rate (WVTR) across the material [5, 19]. The dried scaffolds were mounted on the mouth of a cylindrical test tube (18-mm diameter), which contained 10-ml water. The tube was kept at 37°C and 35% relative humidity in an incubator. The test tube with a scaffold was weighed at regular intervals of time and the weight loss versus time was plotted. WVTR was calculated according to the following formulation:

$$\mathrm{WVTR}=\frac{s\times 24}{A}\left(\frac{\frac{g}{{\;m}^{2}}}{\mathrm{day}}\right),$$

where *A* is the area of the sponge (m^2^) and *S* is slope of the weight loss versus time (g/h).

### Cytotoxicity

Mouse fibroblasts L929 cells were used for the evaluation of cytotoxicity evaluation. L929 cells were maintained in Dulbecco’s modified eagle medium (DMEM, Gibco) supplemented with 10% (v/v) foetal bovine serum and 1% (v/v) penicillin/streptomycin antibiotics. The PSC scaffolds and FSC scaffolds were soaked in culture medium for 24 h at a concentration of 0.1 g/mL, and then the extracts were sterilized with a needle filter (0.22 μm). In each well of 96-well culturing plates, 3000 cells were seeded and cultured with the culture medium or extract medium. The density of L929 cells was determined by the Cell Counting Kit-8 assay (CCK-8, Beyotime, China) after culturing for 12 h, 24 h and 72 h, respectively. After incubating for 1 h by using CCK-8 working solution (1:9) at 37°C, the optic density (OD) of the solution was measured by using a microplate spectrophotometer at 450 nm. The cells cultured with normal culture medium was used as control. Five replicates were considered per scaffold.

### *In vivo* wound healing experiments

The animal experiments were approved by the Animal Research Committee of Huazhong University of Science and Technology. The wound healing activity of each wound dressing was checked on designed burn rabbit model. Two New Zealand White rabbits (weight of 2.5 kg/animal) were used as an animal model in the current study. Inhalation anaesthesia was performed on all animals with isoflurane by using an inhalational anaesthesia machine (RWD Life Science Co., Ltd., Shenzhen). Commercial hair removal cream was used to shave the skin of rabbit back. Thirty-five layers of gauze (2 cm × 2 cm) was boiled in boiling water and then was perpendicularly applied to the skin with a gravitational pressure on the skin for 20 s to induce scald area [20]. There were five wounds each side of each rabbit back, and totally 20 wounds were created. After 24 h, five pieces of PSC scaffolds and five pieces of FSC scaffolds were soaked in saline and were used to cover the wounds in one rabbit. All scaffolds were securely fixed to the rabbit body by commercial breathable non-woven application and elastic adhesive bandage. Similarly, the 10 wounds on the other rabbit was treated by gauze and vaseline gauze, respectively. During these experiments, rabbits were kept in separate cages and fed with commercial foods and water until they were sacrificed. All dressings covered on the wounds were changed once per 3 days. The wounds were grossly examined and photographed for measurement of wound size reduction at 0, 7, 14, 21 and 28 days [21]. The rabbits were euthanized after the experiments. The wound size measurements taken at the time of treatment and at the time of intervals were used to calculate the percentage wound size remaining (*S*) using the formulation:

$$S=\frac{s^{\prime }}{s_{0}}\times 100\%,$$

where $s^{\prime }$ is the remaining wound size after time (*t*) and $s_{0}$ is the initial size. The wound areas were measured from the photographs of the wounds using the image analysis software NIH Image J.

Histological analysis was performed to evaluate the wound healing and tissue regeneration process. Tissue samples were collected from the wounds at 7, 14, 21 and 28 days and fixed in 4% paraformaldehyde for histological analysis. For histological staining, the retrieved samples were processed for sectioning using standard protocols. The sections were stained with H&E and photographed.

### Statistical analysis

All data are expressed as mean ± standard derivation. The *t*-test and one-way ANOVA were used to compare differences between each group. A difference was considered significant when *P* < 0.05.

## Results

### Appearance and porosity

**Figure 1:**
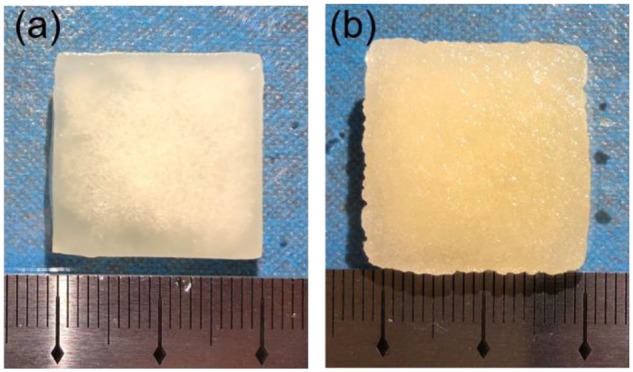
Photographic appearance of the scaffolds (**a**) PSC and (**b**) FSC

The purified collagen powders were prepared by freeze-drying and were dissolved in acidic solution with the same concentration. The obtained solution of PSC seemed thinner than the FSC solution. After freeze-drying, the obtained two scaffolds were put in PBS together. Both scaffolds were very stable and elastic after adsorbing water. The thickness of the scaffolds was about 3–5 mm. They seemed a little different after soaking water, as shown in Fig. 1. The PSC scaffold was smooth, while the FSC scaffold was a little rough in surface. The porosity of the two scaffolds was very high. According to the measurements, the porosity of PSC scaffold was 96.1 ± 2.0%, while the porosity of FSC scaffold was 98.1 ± 0.5%. Such high porosity enabled the in-growth of cells and exchanging nutrients and metabolites.

### Morphology

**Figure 2:**
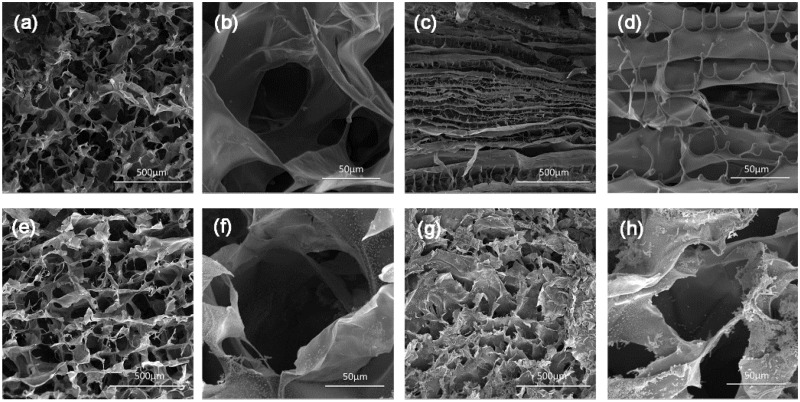
SEM images of the scaffolds (**a**, **b**) top-view of PSC scaffold, (**c**, **d**) vertical cross section of PSC scaffold, (**e**, **f**) top-view of FSC scaffold and (**g**, **h**) vertical cross section of FSC scaffold

The SEM images of the PSC and FSC scaffolds are shown in Fig. 2. The large pores with the diameter about 100–200 μm were observed on both the surface and the cross section of the scaffolds. The porous scaffolds could serve as a matrix for the ingrowth of cells into the wound and promote regeneration. In addition, it was observed that the PSC scaffolds show laminar structures on the cross sections, which were not found in the FSC scaffolds. The possible reason for this difference is the different ice formation during the freezing process. Maybe FSC could retard the ice formation and no laminar structure was formed.

### Composition

**Figure 3:**
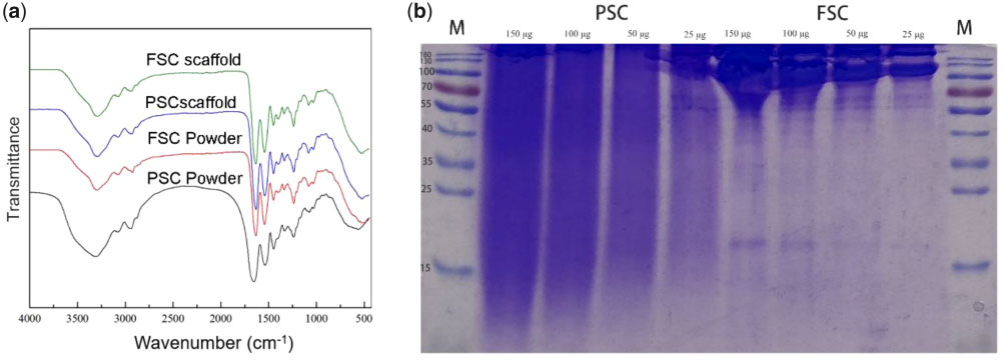
(**a**) FT-IR spectra of PSC and FSC powder and scaffolds and (**b**) SDS-PAGE of PSC and FSC

The composition of the powders and scaffolds was examined by FT-IR, as shown in Fig. 3a. It was suggested that the FT-IR spectrum of FSC shows the same peaks that were also found in PSC. Both collagen materials derived from fish scales and porcine skin are mainly type I collagen. The bands at 3300 cm^−1^ were attributed to the amide A associated with N-H stretching in collagen. The amide B band was observed at 2923–2939 cm^−1^. The bands at 1630–1653 cm^−1^, 1535–1543 cm^−1^ and 1236–1238 cm^−1^ were attributed to amide I, amide II and amide III in collagen, respectively. These typical bands confirmed the collagen structure of both PSC and FSC. The molecular weight of PSC and FSC was also compared by SDS-PAGE, as shown in Fig. 3b. It was found that FSC shows typical regions related to α chain (approximately 100 kDa) and β chain (approximately 200 kDa). Meanwhile, PSC shows wide range on SDS-PAGE results and many small molecules were detected, which suggested a high concentration of degraded collagen in the PSC product.

### Water uptake capacity and vapour transmission

**Figure 4:**
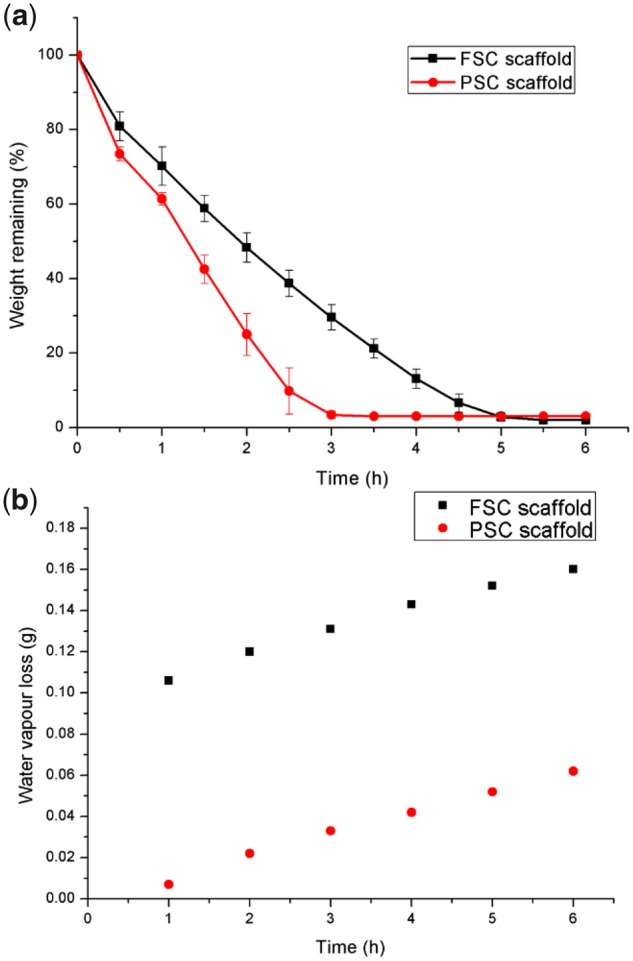
(**a**) Water-holding capacity and (**b**) water vapour transmission loss from the two scaffolds

The water uptake capacity of the wound dressings was investigated. The water uptake ratio of PSC scaffold was 27.4 and that of FSC scaffold reached 47.8. The water preservation ability was evaluated for both scaffolds. According to the weight remaining percentage along the time, it was found that the FSC scaffold lost the water more slowly than the PSC scaffold. After 3 h, PSC scaffold lost all water, while FSC scaffold could last for about 5 h (Fig. 4).

The moist environment around the wounds is very important for a successful wound healing [4]. PSC scaffold showed WVTR of 952.6 ± 55.5 g/mg^2^ day, while FSC scaffold showed a little higher WVTR of 1090.9 ± 77.1 g/m^2^ day. Both scaffolds were a little higher than the commercial Mediafoam dressings (WVTR = 811 g/m^2^ day) [22]. Generally, wound healing could be impeded because of poor drainage of exudation when the WVTR of wound dressing is too low. When the WVTR is too high, the wound surface could become dry due to loss of fluid. In this study, both FSC and PSC scaffolds were considered to perform well in water vapour transmission.

### Cytotoxicity

**Figure 5:**
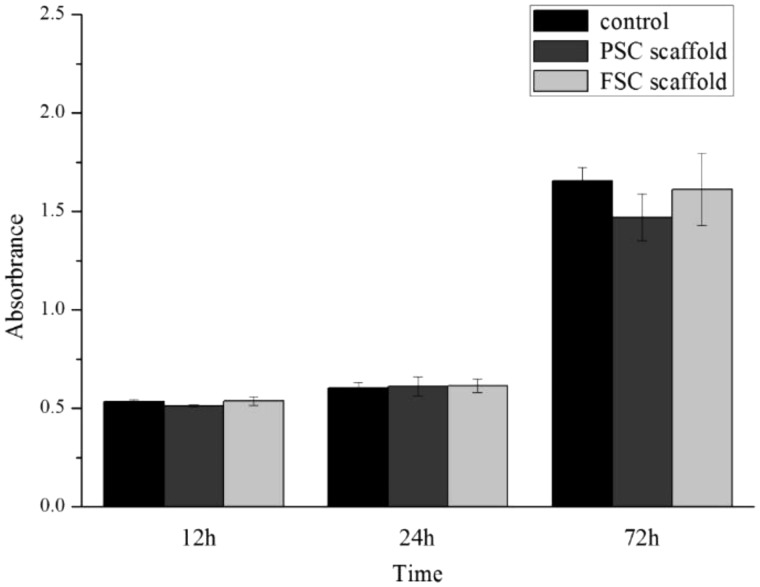
CCk-8 assay results of various extracts of scaffolds along with control

Figure 5 represents CCK-8 assay results on the cell viability and proliferation of L929 cells in the extract medium and control. It was obvious the metabolic activity of L929 cells was not significantly decreased when culturing in the extract medium from the scaffolds after 12 h and 24 h. At 72 h, the OD value of PSC scaffold was about 10% lower than the control and FSC scaffolds. The statistical analysis indicated that no significant difference was found (*P* > 0.05). In general, the toxicity of both PSC and FSC scaffolds is acceptable, which indicated non-toxicity.

### Visual observation of burns and healing process

**Figure 6:**
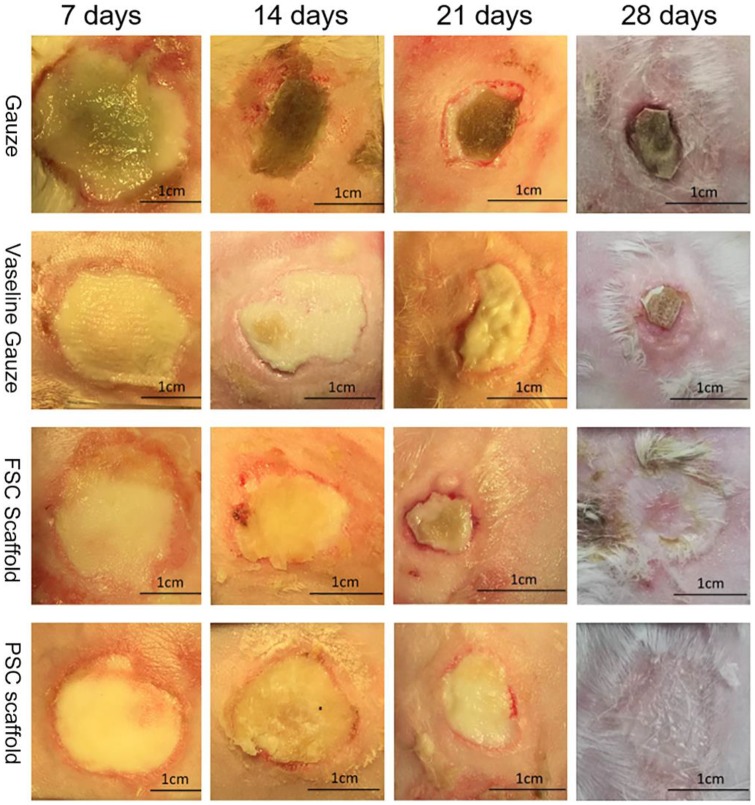
Representative images of burn wounds in gauze, vaseline gauze, FSC scaffold and PSC scaffold groups at days 7, 14, 21 and 28

To compare the wound healing efficiency, PSC and FSC scaffolds were compared to gauze and vaseline gauze, which was used to treat burns in clinic. From Fig. 6, on day 7, the oedema on the wound surface can be seen, especially for the gauze group. On day 14, black scabs formed on the wound of gauze group. The other three groups did not show such obvious scabs. On day 21, the size of the wound area became smaller for all groups. Black scabs were observed for the gauze group. For the vaseline gauze group, only yellow soft tissue was seen. The tissue around the wound recovered well for both PSC and FSC groups. The wound shrinkage seemed better in the FSC scaffold group. On day 28, the wounds in the gauze and vaseline gauze groups had not recovered completely with adherent scabs. During the whole healing process, no scabs were formed for both PSC and FSC groups. In addition, some hair growth was found in the wound area for these two collagen scaffold groups.

**Figure 7:**
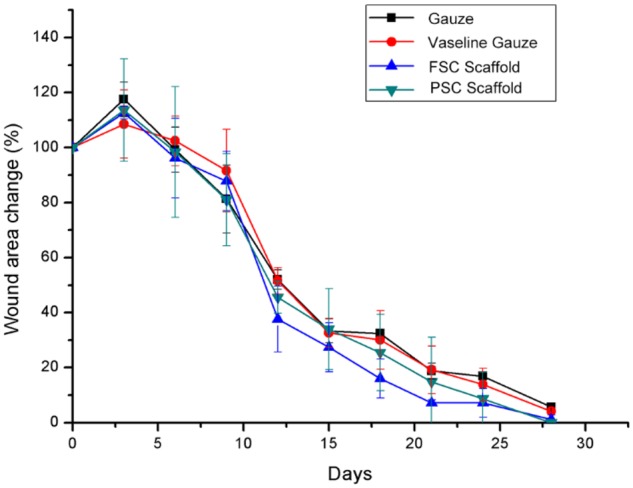
Wound closure area in gauze, vaseline gauze, FSC scaffold and PSC scaffold groups

Figure 7 shows that the reduction rate of wound area was quite similar for the four groups. It was found that the wound areas increased about 20% for the gauze group at day 3, while only 10% for the vaseline gauze group. The area increased about 15% for FSC and PSC scaffolds. It was indicated that vaseline could reduce the wound area at the initial stage of wound recovery. After 9 days, both FSC and PSC show smaller wound areas compared to the vaseline gauze group. For the FSC scaffold, the recovery of the wound seemed a little faster after 12 days than the other three groups. This superiority kept till the wound healed. It can be seen that the healing time of both FSC and PSC is about 28 days. At the endpoint of 28 days, all of the wounds were recovered for both FSC and PSC groups. The wounds treated with gauze and vaseline gauze remained a small unhealed wound percentage, which was consistent with the visual observation.

### Histological examination on healing burns

**Figure 8:**
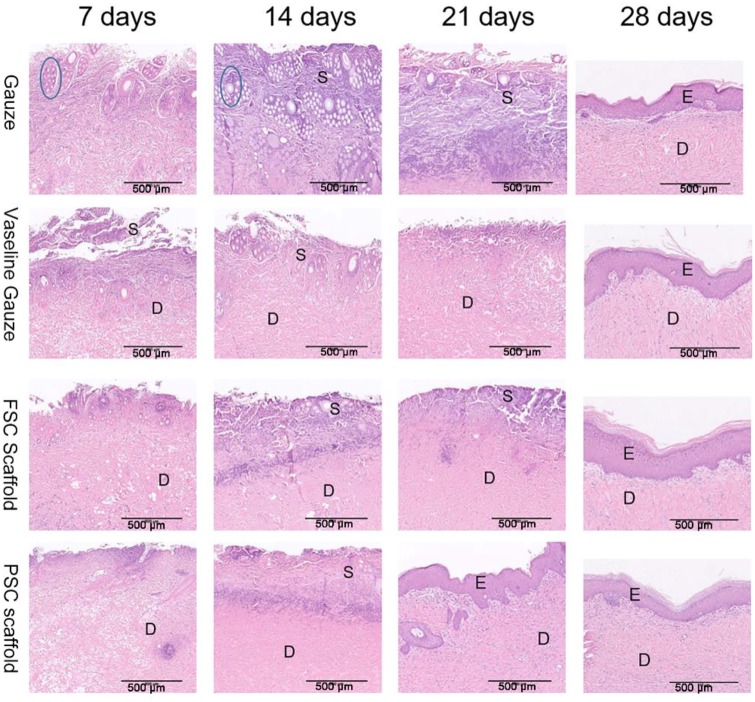
H&E-stained microscopic sections at days 7, 14, 21 and 28

In this study, we conducted a typical deep scald model. As shown in Fig. 8, on day 7, the epidermis was observed damaged. Many blisters in the dermis could be seen in the gauze group. Inflammatory cell infiltration was found in all groups. For the FSC scaffold and PSC scaffold, the tissue recovered quickly and no severe secondary damage was found. On day 14, more inflammatory cells were observed in the gauze group and no epidermis was recovered. For the vaseline gauze group, the change was not very obvious. On day 21, wound recovery was the most obvious in the PSC scaffold group and re-epithelization was almost completed. On day 28, re-epithelization was found for all groups. Combined with the visual observation and histological analysis, both PSC and FSC scaffolds show wound healing acceleration superiority compared to gauze and vaseline gauze.

## Discussion

Healing of burn wound depends on the severity of burns and the subsequent treatments. Deep dermal burn injury is complicated, and it will result in ugly scars if the treatment is not suitable. For the manufactures of the advanced wound dressings, collagen is extensively used as porous sponge or scaffolds when treating such complicated wounds [2, 6]. In the present study, we successfully fabricated two kinds of collagen sponge-like scaffolds using PSC and FSC, respectively. Their morphology indicated a highly porous structure and the porosity was very high. The ability to adsorb extrude during the inflammatory phase is crucial for wound dressing materials; therefore, the swelling properties of the dressings were evaluated. It was found the FSC scaffold could uptake 47.8 times water of its weight, which is higher than the PSC scaffold (27.4 times). Besides this high swelling ratio, the collagen scaffolds can let the water evaporate with a suitable rate. The WVTR of both scaffolds could meet the demands of an optimally moist environment. The moist wound healing was also approved by the visual observation and histological analysis. The burn wound recovered faster and the final appearance was better than the control groups. Overall, the *in vivo* study showed that the healing capacity of collagen scaffolds was superior to gauze and vaseline gauze groups. It was suggested that FSC could be used instead of PSC, which reduced the risk of zoonosis and could be beneficial for clinical applications. In addition, the fish scales are a much cheaper and safer source of collagen, as they are a waste product thrown away.

Besides burn, collagen-based wound dressings have been used to treat diabetes-related foot ulcers [23]. Many chronic wounds could be treated using collagen-based dressings [4, 9, 24]. Herein, we found that the FSC scaffolds could perform similar with the PSC scaffolds in the burn wound model. In seemed that the FSC scaffolds would be a promising candidate for the applications in other chronic wounds. Furthermore, collagen can be blended with other polymers to adjust the properties, including mechanical strength and antibacterial properties [4, 23, 25, 26]. In addition, nanofibers prepared by electrospinning could be applied to collagen-based materials, and advanced wound dressings composed of nanofibers have been developed as controlled delivery system for drugs and growth factors [27–29]. In the previous study, plant extract was incorporated into FSC sponge to enhance the wound healing effect [30]. Composite electrospun nanomembranes containing FSC and chito-oligosaccharide were found to show good anti-bacterial activity and biocompatibility [31]. It was suggested that FSC could be used as an alternative of mammalian tissue derived collagen in many applications.

## Conclusion

Sponge scaffolds for burn healing have been developed by using PSC and FSC, respectively. Both scaffolds are highly porous and the PSC scaffolds show laminar microstructure. The FSC scaffold has higher water-uptake, slower water loss and higher WVTR compared to PSC scaffold. Based on the macroscopic and histological examinations, both PSC and FSC could significantly promote burn healing, showing good wound-healing outcome compared to gauze and vaseline gauze groups. It was concluded that FSC could be used as an alternative candidate in burn wound caring applications as well as PSC.
